# An Investigation of the Relationship Between Dietary Patterns in Early Pregnancy and Maternal/Infant Health Outcomes in a Chinese Cohort

**DOI:** 10.3389/fnut.2022.775557

**Published:** 2022-04-22

**Authors:** Jamie V. de Seymour, Kathryn L. Beck, Cathryn A. Conlon, Mary Beatrix Jones, John Colombo, Yin-Yin Xia, Ting-Li Han, Hong-Bo Qi, Hua Zhang, Philip N. Baker

**Affiliations:** ^1^College of Health, Massey University, Auckland, New Zealand; ^2^Department of Statistics, University of Auckland, Auckland, New Zealand; ^3^Department of Psychology and Schiefelbusch Institute for Life Span Studies, University of Kansas, Lawrence, KS, United States; ^4^School of Public Health and Management, Chongqing Medical University, Chongqing, China; ^5^Department of Obstetrics and Gynaecology, The First Affiliated Hospital of Chongqing Medical University, Chongqing, China; ^6^College of Life Sciences, University of Leicester, Leicester, United Kingdom

**Keywords:** maternal diet, neurocognition, infant outcomes, dietary patterns, Bayley Scales of Infant Development, gestational weight gain, placenta, infant skinfolds

## Abstract

**Background:**

Studies assessing links between maternal diet and pregnancy outcomes have focused predominantly on individual nutrients or foods. However, nutrients are typically consumed in combinations of foods or beverages (i.e., dietary patterns). Taking into account the diet as a whole appreciates that nutrient absorption and metabolism are influenced by other nutrients and the food matrix.

**Objective:**

The aim of this study was to investigate the relationship between dietary pattern consumption in early pregnancy and pregnancy/infant outcomes, including gestational diabetes mellitus, gestational weight gain, preeclampsia, placental weight, gestational age at delivery, small-for-gestational-age, large-for-gestational-age, macrosomia, measures of infant body composition, and scores on two main indices of the Bayley Scales of Infant Development [Mental Development Index (MDI) and the Psychomotor Development Index (PDI)] at 12 months.

**Design:**

Our study included 1,437 participants from a mother-infant cohort in Chongqing, China. Maternal diet was assessed using a 96-item food frequency questionnaire at 11–14 weeks gestation. Dietary patterns were constructed using principal component analysis. Multivariate regressions were performed to assess associations between maternal dietary pattern scores and pregnancy and infant outcomes, adjusting for confounders.

**Results:**

Two dietary patterns were derived: a pattern high in pasta, sweetened beverages, and oils and condiments (PSO-based dietary pattern) and a pattern high in fish, poultry, and vegetables (FPV-based dietary pattern). Higher scores on the PSO-based dietary pattern were associated with lower infant standardized scores on the PDI of the Bayley Scales of Infant Development, β (95% confidence interval) = −1.276 (−2.392, −0.160); lower placental weight, β (95% CI) = −6.413 (−12.352g, −0.473); and higher infant's tricep skinfold thickness at 6 weeks of age. β (95% CI) = 0.279 (0.033, 0.526). Higher scores on the FPV-based dietary pattern were associated with higher gestational weight gain between visit 1 (11–14 week's gestation) and 3 (32–34 week's gestation). β (95% CI) = 25.612 (13.255, 37.969). No significant associations were observed between dietary pattern scores and the remaining pregnancy/infant outcomes investigated or MDI scores on the Bayley Scales of Infant Development. This was the first study to investigate the association between dietary patterns in early pregnancy and infant neurocognition in a Chinese cohort.

## Introduction

Maternal diet is widely accepted as an influential factor in the maintenance of a healthy pregnancy and the prevention of some pregnancy complications (1, 2). However, relationships between maternal diet and pregnancy outcomes differ between studies and across populations (3). One aspect of these inconsistencies may lie in the methodological approaches that are employed to examine diet-disease relationships. These include differences in how the diet is investigated (i.e., studies investigating associations with individual nutrients, supplementation, or dietary pattern consumption), different timepoints in pregnancy that the diet is studied, how dietary assessment is conducted (i.e., some studies use food frequency questionnaires, while others use 24-h dietary recalls, or food diaries capturing intake over multiple days), varying approaches in deriving dietary patterns (i.e., *a-priori* or *a-posteriori* approaches), and differing measures of pregnancy outcomes (i.e., the different tests used to assess infant neurodevelopment).

Many studies investigating the links between maternal diet and pregnancy outcomes have studied individual nutrient intake, nutrient supplementation, or individual foods. In reality, however, nutrients or foods are not consumed in isolation. In humans, nutrients are consumed in most instances (with the exception of nutrient supplementation) in foods or beverages. Foods and beverages are eaten in combinations to form meals and the combination of meals consumed over a period of time is referred to as a dietary pattern (4). This is an important concept, as it has been shown that nutrients can act synergistically (5). Taking into account the diet as a whole is more representative of the actual intake of a population and appreciates that nutrients are not consumed by themselves, nor do they act as individual components, being influenced by other nutrients and the food matrix in which they are consumed. In recent years there has been a shift toward dietary pattern analysis to investigate associations between diet and disease. Both *a priori* and *a posteriori* approaches have been employed (6).

Studies investigating the relationships between maternal dietary patterns and pregnancy outcomes have predominantly studied preterm birth, birthweight, gestational diabetes mellitus (GDM), and preeclampsia (7–11). The majority of these studies have been conducted in Western populations, and have emphasized the benefits of consuming a “prudent” type dietary pattern (e.g., high in vegetables, fruit and berries, nuts, whole grains, poultry, seafood and eggs) and the increased risks associated with consuming a “western” type dietary pattern (e.g., high in refined grains, processed and red meat, animal fat (butter and lard), high-fat dairy, salty and sweet snacks) (7–9). These findings are relevant for the populations from which they are derived. But the extrapolation of such findings to populations with vastly different cultures, food availability, and eating styles (e.g., Asian populations) may be questionable. As an example of this, dietary patterns found to be associated with GDM in a large Singaporean cohort of pregnant women (10) characterized by high intakes of seafood, soup, fish and seafood products, and noodles was found to be associated with a reduced risk of GDM. This pattern differed greatly from dietary patterns previously reported in Western populations to be associated with GDM (12–14). No studies to date have focused on the relationship between maternal dietary patterns and infant neurodevelopment in an Asian context.

The current study included participants from a large mother-infant cohort in Chongqing, China to investigate the relationship between dietary patterns in early pregnancy and pregnancy/infant outcomes, including GDM, gestational weight gain, preeclampsia, placental weight, gestational age at delivery, small-for-gestational-age (SGA), large-for-gestational-age (LGA), macrosomia, measures of infant body composition, and infant neurodevelopment at 12 months.

## Materials and Methods

### Study Participants

Participants from the Complex Lipids in Mothers and Babies (CLIMB) cohort were enrolled into this study (Clinical trial registration: ChiCTR-IOR-16007700). Detailed information about the CLIMB study has been published elsewhere (15, 16). Participants were eligible for the CLIMB study if they were between 20 and 40 years of age and were at 11–14 weeks gestation with a singleton pregnancy. Mothers were excluded from the study if they had an allergy to milk protein, severe lactose intolerance, milk aversion, or a previous pregnancy complication resulting in delivery before 32 weeks of gestation. CLIMB participants were eligible for the current study if they completed a food frequency questionnaire (FFQ) at their first visit (between 11 and 14 weeks gestation). The CLIMB study included a total of 1,500 participants and the intervention did not have any significant effect on maternal or infant outcomes, including infant cognitive outcomes (17). The dietary patterns analyzed in the current study were not part of the intervention. All analyses adjusted for the intervention as a precautionary measure, to ensure that any associations observed were not confounded by the intervention.

Ethical approval for this study was granted by the Ethics Committee of Chongqing Medical University (#2014034). The study was conducted in accordance with the principles in the Declaration of Helsinki 1964 and the International Conference on Harmonization Good Clinical Practice E6 (ICH-GCP), and with all applicable regulatory requirements. All participants provided written informed consent.

### Dietary Pattern Analysis

Participants completed a 96-item food frequency questionnaire (FFQ) at 11–14 weeks gestation, which reflected their dietary intake over the previous 3 months. This FFQ was an adaptation of the FFQ used in the S-PRESTO study in Singapore (18) to include foods relevant to the Chongqing context. The 96 items in the FFQ were condensed into 28 food groups based on similarity of nutrient content and on food groupings previously published in studies of dietary patterns in pregnant Chinese women (11, 19, 20). Exploratory factor analysis with varimax rotation was performed to derive dietary patterns from the 28 food groups. The number of factors (dietary patterns) to consider was determined by the elbow in the scree plot and interpretability of the patterns produced. Food groups with factor loadings above 0.25 were used to describe the patterns and the patterns were named after food groups with a factor loading above 0.40. All participants received a factor score for each of the dietary patterns identified, using the regression method. A higher score indicated higher adherence to that dietary pattern.

### Pregnancy and Infant Outcome Data

Participants attended four research visits at the First Affiliated Hospital of Chongqing Medical University and Chongqing Health Centre for Women and Children during their pregnancy and two further visits after their delivery: 11–14 weeks' gestation (visit 1), 22–28 week's gestation (visit 2), 32–34 week's gestation (visit 3), at birth (visit 4), 6 weeks postnatal (visit 5), and 12 months postnatal (visit 6). Details of the assessments undertaken at each visit have been previously described (15). Participants underwent a 75 g oral glucose tolerance test (OGTT) at 22–28 week's gestation. Socio-demographic data was collected at enrolment through interviews with trained nurses, including maternal age, ethnicity, education level (categorized as: tertiary level education or below), household income level (categorized as: <7,000 yuan/month, 7,000–10,000 yuan/month, or >10,000 yuan/month), and body mass index (BMI) was measured at the first visit and calculated as weight (kg)/height (m)^2^. Gestational age was calculated using the last menstrual period and confirmed with ultrasound dating. In the case of the two gestational age estimates being discordant by more than 7 days, the ultrasound date was used.

Gestational diabetes mellitus (GDM) was diagnosed following the OGTT, according to the International Association of Diabetes and Pregnancy Study Groups (IADPSG) criteria: fasting glucose ≥ 5.1 mmol/l, 1 h glucose ≥ 10 mmol/l, OR 2 h glucose ≥ 8.5 mmol/l (21). Gestational weight gain was calculated as the weight gain between visit 1 and visit 3, as an average in grams/week. Preeclampsia was diagnosed by the participant's leading maternity care providers using the following criteria: systolic blood pressure >140 mmHg and/or diastolic blood pressure >90 mmHg on at least two occasions 4 h apart after 20 weeks of gestation, with significant proteinuria (spot urine protein/creatinine >30 mg/ mmol [0.3 mg/mg] or >300 mg/day or at least 1g/L [“2 +”] on dipstick testing) (22).

Data on placental weight, birthweight, and gestational age at delivery were collected at visit 4. Babies were considered SGA or LGA based on the 10 and 90th centiles of birth weight for gestation, using birthweight references specific to Chongqing (23). Babies met the criteria for macrosomia if they had a birthweight above 4,000 g. Ponderal index was calculated using length and weight at birth with the following equation: weight (g)/length (cm)3. Infant's subscapular and tricep skinfold thickness, and mid-arm circumference was measured by trained research staff at visit 5.

### Assessment of Infant Neurodevelopment

Infant neurodevelopment was assessed at visit 6 (between 11.5 and 12.5 months of age) using the Chinese version of the Bayley Scales of Infant Development (BSID) (24). The Chinese version of the BSID is a formal adaptation to the Chinese language and locally standardized to reflect culturally appropriate scoring on two main indices: the Mental Development Index (MDI) and the Psychomotor Development Index (PDI). The MDI component comprised 163 items and assessed age-appropriate items related to cognitive functioning, personal and social development, and language development. The PDI component comprised 81 items and assessed age-appropriate fine and gross motor skills. The test provided raw scores for both indices which were transformed to standardized scores based on norms for the Chinese population. The resulting standardized index scores had a mean of 100 and a standard deviation of 15, with a lower score reflecting poorer performance.

### Statistical Analysis

All statistical analyses were performed using SPSS v26.0; *p*-values < 0.05 were considered statistically significant. Non-normal data were reported as medians [lower quartile (LQ), upper quartile (UQ)] and Mann Whitney-U tests were performed to assess differences between groups. Normally distributed data were reported as mean (± standard deviation) and Student *t*-tests were performed to assess differences between groups. Chi-square tests were performed on categorical variables to test for differences between groups. Macronutrient and energy (kcal) intakes were reported according to tertiles of dietary pattern scores.

Multivariate linear regression was used to assess relationships between dietary pattern scores and continuous pregnancy and infant outcomes. Binomial logistic regression was used to assess relationships between dietary pattern scores and binary pregnancy and infant outcomes. Unadjusted models and adjusted models were established. Eight variables were included in the adjusted models. Variables were selected for inclusion in the models based on epidemiological evidence of having a relationship either with the exposure, outcome, or both the exposure and the outcome. The following variables were included in the model as covariates: maternal age (at visit 1), offspring sex, and energy intake. The following variables were included in the models as potential confounders: maternal BMI (at visit 1), CLIMB treatment group, maternal education level, family income, and maternal ethnicity.

## Results

### Study Participants

A total of 1,499 women who were enrolled in the CLIMB study completed a first trimester food frequency questionnaire (FFQ). Participants with a total volume of food intake above or below 2 standard deviations of the mean (grams) were deemed outliers (*N* = 62) and not included in further analysis. Therefore, a total of 1,437 women were included in this study. Participant characteristics are displayed in Table 1. Participants had a median [lower quartile (LQ), upper quartile (UQ)] age of 28 years (26, 31) and a median (LQ, UQ) BMI of 21.0 kg/m2 (19.4, 22.9), 63.1% had completed tertiary education, 97.9% were of Han Chinese ethnicity, and 48.6% of the babies born were female.

**Table 1:** Participant (maternal and infant) characteristics.

**Characteristic**	
**Maternal**	
Age (years)	28 (26,31)
Body mass index (kg/m^2^)	21.0 (19.4, 22.9)
**Han ethnicity**	
Yes	1,407 (97.9%)
No	30 (2.1%)
**Tertiary-level education**	
Yes	903 (63.1%)
No	527 (36.9%)
**Household income**	
<7,000 yuan/month	279 (19.5%)
7,000–10,000 yuan/month	514 (35.9%)
>10,000 yuan/month	637 (44.5%)
**Gestational diabetes mellitus**	
Yes	349 (27.7%)
No	912 (72.3%)
Gestational weight gain (g/week)	8.9 (3.8)
**Preeclampsia**	
Yes	21 (1.7%)
No	1,201 (98.3%)
**Infant**	
Gestational age at delivery (weeks)	39.6 (38.9, 40.3)
**Sex**	
Male	642 (51.4%)
Female	607 (48.6%)
Birthweight (g)	3,303 (433)
Placental weight (g)	550 (510, 600)
**Small-for-gestational-age (SGA)**	
Yes	47 (3.8%)
No	1,184 (96.2%)
**Large-for-gestational-age (LGA)**	
Yes	121 (9.8%)
No	1,110 (90.2%)
**Macrosomia**	
Yes	59 (4.8%)
No	1,172 (95.2%)
Ponderal index (kg/m^3^)	2.67 (2.53, 2.81)
Subscapular skinfold thickness (mm)	7.90 (6.80, 9.50)
Tricep skinfold thickness (mm)	12.99 (2.55)
Mid-arm circumference (mm)	13.22 (1.28)
**Bayley Scales of Infant Development**	
Psychomotor development index standardized score	86 (77, 96)
Mental development index standardized score	95 (84, 106)

*Non-normal data were reported as medians [lower quartile (LQ), upper quartile (UQ)] and normally distributed data were reported as mean (± standard deviation)*.

### Dietary Pattern Analysis

**Figure 1:**
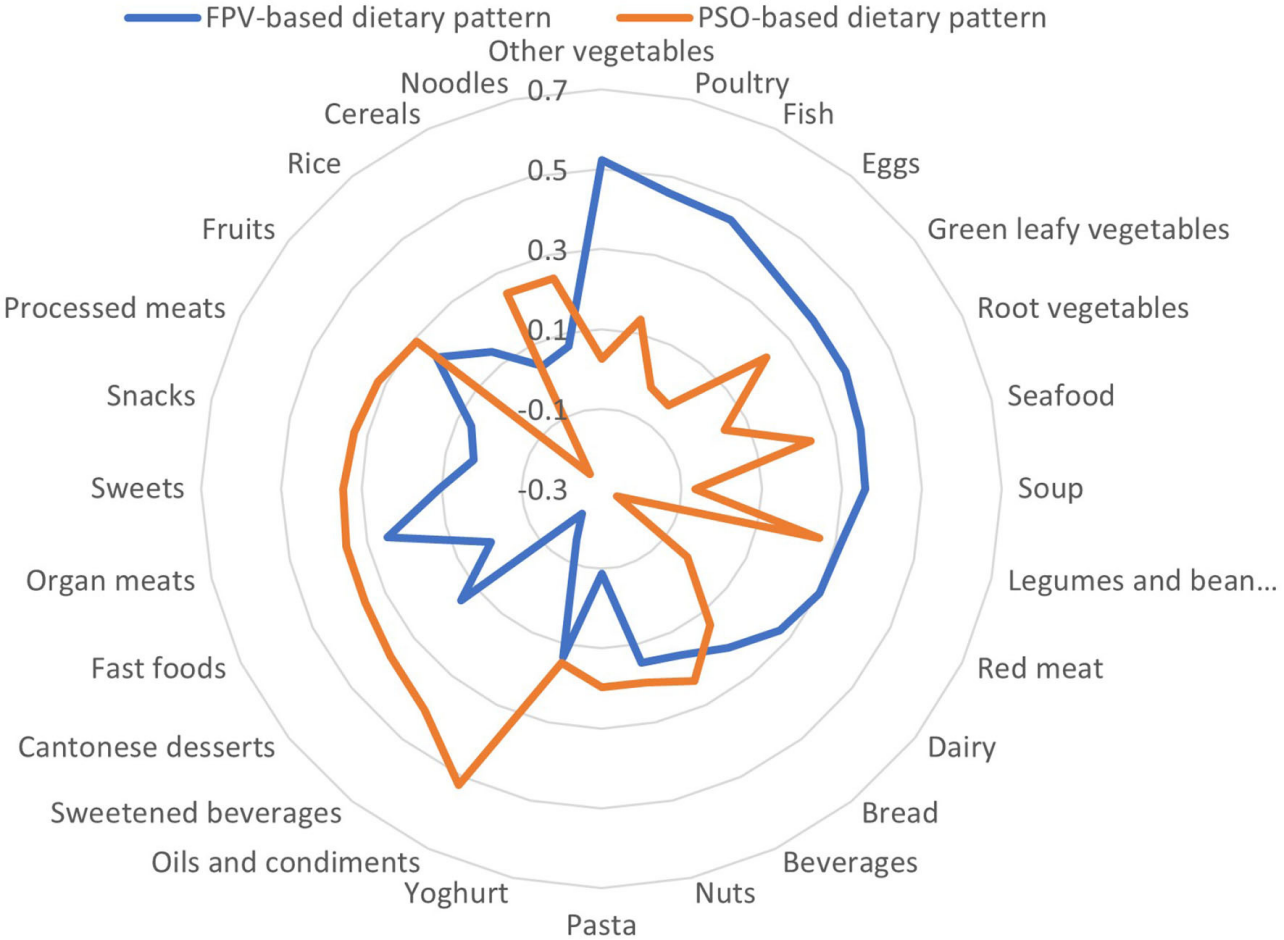
Radar plot of the factor loadings of the 28 food groups on the two dietary patterns.

Two main dietary patterns were found to be consumed in the cohort: a fish, poultry and vegetables (FPV)-based dietary pattern and a pasta, sweetened beverages, oils and condiments (PSO)-based dietary pattern (Table 2, Figure 1). The two main dietary patterns together explained 13.9% of the total variation in dietary intake.

**Table 2:** Key food components contributing to the two main dietary patterns consumed in the CLIMB cohort.

**Fish, poultry, and vegetables (FPV)–based dietary pattern** **8.4% variance explained**		**Pasta, sweetened beverages, oils and condiments (PSO)-based dietary pattern** **5.9% variance explained**	
**Food group**	**Factor loading**	**Food group**	**Factor loading**
Other vegetables	0.522	Oils and condiments	0.769
Poultry	0.453	Pasta	0.654
Legumes and bean products	0.444	Sweetened beverages	0.512
Green leafy vegetables	0.428	Fast food	0.258
Fish	0.426		
Root vegetables	0.380		
Seafood	0.352		
Fruits	0.351		
Eggs	0.331		
Organ meats	0.291		
Beverages	0.282		
Bread	0.277		
Dairy	0.258		
Soup	0.254		
Nuts	0.253		

*Food groups with a factor loading above 0.25 or below −0.25 are displayed. FPV- Fish, poultry, and vegetables; PSO- Pasta, sweetened beverages, oils and condiments*.

The energy intake and macronutrient distribution across tertiles of dietary pattern scores are displayed in Table 3. Women scoring in the top tertile of the FPV-based dietary pattern consumed a higher number of total kilocalories than those in the lowest and middle tertiles, whereas the opposite was observed in the PSO-based dietary pattern tertiles. Top tertile scorers on the FPV-based dietary pattern also consumed a higher amount of protein and fat as a percentage of total kilocalories, than participants in the other tertiles, whereas those scoring in the top tertile of the PSO-based dietary pattern consumed a higher amount of carbohydrates as a percentage of total kilocalories than participants in the other two tertiles.

**Table 3:** Energy and macronutrient consumption of participants according to tertiles of dietary pattern scores.

		**Energy (kcal)**	**Carbohydrate (% of energy)**	**Fat (% of energy)**	**Protein (% of energy)**
		**Median (LQ, UQ)**	**Median (LQ, UQ)**	**Median (LQ, UQ)**	**Median (LQ, UQ)**
FPV-based dietary pattern	Low	2,286 (1,715, 2,612)	75.2 (69.8, 79.9)	14.6 (11.1, 18.2)	11.8 (10.9, 12.9)
	Medium	2,568 (2,182, 2,921)	72.3 (67.8, 76.3)	16.6 (13.3, 20.2)	12.6 (11.9, 13.7)
	High	2,853 (2,462, 3,367)	69.2 (64.9, 73.0)	18.3 (15.8, 21.5)	13.9 (12.8, 15.1)
PSO-based dietary pattern	Low	2,752 (2,391, 3,055)	70.2 (65.6, 74.7)	17.6 (14.6, 21.5)	12.9 (12.0, 14.2)
	Medium	2,457 (2,067, 2,829)	72.7 (67.9, 77.1)	16.0 (12.3, 19.7)	12.6 (11.6, 13.9)
	High	2,473 (1,928, 2,892)	72.8 (67.7, 77.2)	16.5 (13.2, 20.1)	12.7 (11.6, 13.9)

*LQ, lower quartile; UQ, upper quartile; FPV, Fish, poultry, and vegetables; PSO, Pasta, sweetened beverages, oils and condiments*.

### Relationship Between Dietary Pattern Scores, Pregnancy and Infant Outcomes

A negative linear association was found between the PSO-based dietary pattern scores and placental weight (Table 4), in the adjusted model (6.413 g reduction in placental weight per SD increase in PSO score, 95% CI: −12.352, −0.473 g). Higher scores on the PSO-based dietary pattern were associated with lower infant standardized scores on the PDI (Table 5), in both an unadjusted model (1.231 point reduction on the standardized PDI per SD increase in PSO score, 95% CI: −2.337, −0.126 points) and an adjusted model (1.276 point reduction on the standardized PDI per SD increase in PSO score, 95% CI: −2.392, −0.160 points).

**Table 4:** Linear regression analyses of dietary pattern scores and pregnancy outcomes.

	**β (95% CI)**	***P*** **-value**
**Placental weight (*****N*** **= 1,015)**		
**FPV-based dietary pattern**		
Unadjusted model	−3.030 (−7.470, 1.411)	0.181
Adjusted model	−0.449 (−5.714, 4.816)	0.867
**PSO-based dietary pattern**		
Unadjusted model	−1.965 (−6.145, 2.214)	0.356
Adjusted model	−6.413 (−12.352, −0.473)	0.034*
**Gestational weight gain (*****N*** **= 1,193)**		
**FPV-based dietary pattern**		
Unadjusted model	24.437 (13.796, 35.078)	<0.001*
Adjusted model	25.612 (13.255, 37.969)	<0.001*
**PSO-based dietary pattern**		
Unadjusted model	9.991 (−2.956, 22.938)	0.130
Adjusted model	6.772 (−6.342, 19.886)	0.311

Models adjusted for CLIMB treatment group, offspring sex, mother's education level, age and BMI, family income, ethnicity, energy intake, and other dietary pattern. CI, confidence intervals; FPV, Fish, poultry, and vegetables; PSO, Pasta, sweetened beverages, oils and condiments. ^*^P < 0.05.

**Table 5:** Linear regression analyses of dietary pattern scores and infant outcomes.

	**β (95% CI)**	***P*** **-value**
**Standardized scores on the Bayley Scales of Infant Development Psychomotor development index at 12 months (*****N*** **= 974)**		
**FPV-based dietary pattern**		
Unadjusted model	−0.266 (−1.214, 0.683)	0.583
Adjusted model	−0.368 (−1.423, 0.687)	0.494
**PSO-based dietary pattern**		
Unadjusted model	−1.231 (−2.337, −0.126)	0.029*
Adjusted model	−1.276 (−2.392, −0.160)	0.025*
**Standardized scores on the Bayley Scales of Mental Development Index at 12 months (*****N*** **= 974)**		
**FPV-based dietary pattern**		
Unadjusted model	−0.884 (−1.993, 0.225)	0.118
Adjusted model	−0.344 (−1.570, 0.882)	0.582
**PSO-based dietary pattern**		
Unadjusted model	−0.582 (−1.874, 0.710)	0.377
Adjusted model	−0.424 (−1.721, 0.873)	0.521
**Tricep skinfold thickness at 6 weeks (*****N*** **= 744)**		
**FPV-based dietary pattern**		
Unadjusted model	0.031 (−0.161, 0.222)	0.755
Adjusted model	0.014 (−0.200, 0.228)	0.897
**PSO-based dietary pattern**		
Unadjusted model	0.281 (0.036, 0.527)	0.025*
Adjusted model	0.279 (0.033, 0.526)	0.027*

Models adjusted for CLIMB treatment group, offspring sex, mother's education level, age and BMI, family income, ethnicity, energy intake, and other dietary pattern. CI, confidence intervals; FPV, Fish, poultry, and vegetables; PSO, Pasta, sweetened beverages, oils and condiments. ^*^P < 0.05.

A positive linear relationship was observed between scores on the FPV-based dietary pattern and gestational weight gain between visit 1 (11–14 week's gestation) and 3 (32–34 week's gestation) (Table 4) in an unadjusted model (24.437 g increase in weight gain/week per SD increase in FPV score, 95% CI: 13.796, 35.078 g/week), which remained significant after adjustment for confounding variables and covariates (25.612 g increase in weight gain/week per SD increase in FPV score, 95% CI: 13.255, 37.969 g/week).

Higher FPV-based dietary pattern scores were associated with an increased likelihood of LGA, however, the association did not remain significant after adjustment for confounders (Supplementary Table 1). Scores on the PSO-based dietary pattern were positively associated with the infant's tricep skinfold thickness at 6 weeks of age, in an unadjusted model (0.281 mm increase in tricep skinfold thickness per SD increase in PSO score, 95% CI: 0.036, 0.527 mm), and this relationship remained significant in an adjusted model (0.279 mm increase in tricep skinfold thickness per SD increase in PSO score, 95% CI: 0.033, 0.526 mm) (Table 5).

No significant associations were observed between the dietary pattern scores and infant's standardized scores on the MDI (Table 5), gestational age at delivery, odds of preterm birth, preeclampsia, GDM, SGA, or macrosomia, infant subscapular skinfolds, or mid-arm circumference (Supplementary Tables 1, 2).

### Sensitivity Analysis

All participants included had biologically plausible dietary intakes from each of the food groups, but as expected with a large sample size, a very broad range of dietary intakes was observed. Therefore, to test whether relationships observed held consistently across the range of intakes, or were driven by the most extreme observations, a sensitivity analysis was undertaken. Participants with the highest and lowest 0.5% of scores on each of the dietary patterns were removed, and the data reanalyzed with the remaining 1,409 participants. Results of the sensitivity analysis are displayed in Supplementary Tables 3, 4. The relationships between the PSO-based dietary pattern and placental weight and PDI scores were no longer statistically significant in the sensitivity analysis, although a strong trend remained (Adjusted *P* = 0.079 and Adjusted *P* = 0.085, respectively). Gestational weight gain remained significantly related to scores on the FPV-based dietary pattern after adjustment for confounding variables and covariates. The relationship between FPV-based dietary pattern scores and increased risk of LGA became significant in the adjusted model as well as the unadjusted model, and there was a borderline significant relationship between FPV-based dietary pattern scores and risk of macrosomia (*P* = 0.045).

## Discussion

In this study we identified two main dietary patterns consumed in our cohort of Chinese mothers, in early pregnancy: a pattern high in pasta, sweetened beverages, and oils and condiments (PSO-based dietary pattern) and a pattern high in fish, poultry, and vegetables (FPV-based dietary pattern). Higher scores on the PSO-based dietary pattern were associated with lower infant scores on the PDI at 12 months of age, lower placental weight, and higher infant's tricep skinfold thickness at 6 weeks of age. Whereas, higher scores on the FPV-based dietary pattern were associated with higher gestational weight gain between visit 1 (11–14 week's gestation) and 3 (32–34 week's gestation). No significant associations were observed between dietary pattern scores and the remaining pregnancy/infant outcomes investigated.

### Maternal Dietary Patterns and Infant Neurodevelopment at 12 Months

This study is the first to report an association between dietary patterns consumed during early pregnancy and infant neurodevelopment in a Chinese cohort. We found that consumption of a dietary pattern higher in pasta, sweetened beverages, oils and condiments, and fast food (PSO-based dietary pattern) was associated with a lower infant PDI score on the Bayley Scales of Infant Development at 12 months, even after controlling for variables that reflect family socioeconomic status. Scores on the PDI at 12 months are a good index of the infant's early motor function, reliably indicating where a child is at a particular time, relative to well-standardized norms. Despite observing a significant association between the PSO-based dietary pattern scores and infant PDI scores, no associations were found between the maternal dietary patterns and infant MDI scores. A study of maternal docosahexaenoic acid supplementation for 4 months postpartum (while breastfeeding) found that maternal DHA exposure was associated with higher PDI but not MDI scores of the infants at 30 months of age (25). Therefore, our study is not the first to find that maternal diet is associated with infant psychomotor development, without significantly influencing mental development.

Very few studies have investigated the relationship between maternal dietary patterns and infant neurodevelopment. One study published in 2022 investigated the relationship between maternal diet quality during pregnancy and its association with infant cognition in 1,580 mother–child pairs in a US cohort (26). The authors assessed diet quality using the Mediterranean Diet Score (MDS-P) and Alternate Healthy Eating Index (AHEI-P) and measured infant neurodevelopment using standardized tests in infancy (median age: 6.4 mo; range: 5.2–10.0 mo), early childhood (median age: 3.2 y; range: 2.8–6.2 y), and mid-childhood (median age: 7.7 y; range: 6.6–10.9 y). It was found that mothers scoring in the highest quartile of the AHEI-P, compared to the lowest quartile, had children with higher Wide Range Assessment of Visual Motor Abilities matching scores in early childhood. They also found that mothers scoring highly on the MDS-P in the first and second trimesters of pregnancy had children with higher Kaufman Brief Intelligence Test, second edition verbal and non-verbal scores in mid-childhood. However, no associations were found between maternal diet quality and cognition at the infancy visit. A study conducted in the UK [Avon Longitudinal Study of Parents and Children (ALSPAC)] on 6,979 mother-children pairs also found that diet quality during pregnancy (18–32 weeks' gestation) was associated with cognitive outcomes of their offspring at eight years of age, as assessed using performance and verbal intelligence quotient assessments of the Wechsler Intelligence Scale for children (27). In our study, we observed a significant association between dietary pattern consumption in early pregnancy and psychomotor development at 12 months, an aspect of infant neurodevelopment that was not captured in the assessments performed by Mahmassani et al. (26) at their infancy visit (only a Verbal Recognition Memory test was administered). However, the findings from the aforementioned studies suggest that associations between maternal diet quality during pregnancy and child neurodevelopment (both cognitive and motor development) may also be observed later in childhood.

A separate study was conducted using >6,500 mother-child pairs from the ALSPAC cohort, to investigate the relationship between maternal dietary patterns and infant intelligence quotients at eight years of age (28). *K*-means clustering was performed on food frequency questionnaire data collected at 32 week's gestation. Three clusters were identified: a “fruit and vegetables” cluster, “meat and potatoes” cluster, and a “white bread and coffee” cluster. The children's IQ was measured at eight years of age using the Wechsler Intelligence Scale for Children. Children of women in the “meat and potatoes” cluster and “white bread and coffee” cluster had lower verbal, performance, and full-scale IQ when compared to children of mothers in the “fruit and vegetables” cluster (28). The only study, to our knowledge, which has investigated dietary patterns of Chinese pregnant women and their relationship with infant's neurodevelopment, was published in 2022 (29). Lv et al. (29) performed dietary pattern analysis on maternal dietary data of 1,178 pregnant women, collected in the second and third trimesters, using a semiquantitative FFQ. Four dietary patterns were identified: an “Aquatic products and Homonemeae” pattern and “Nut” pattern, which were observed in both trimesters; a “Haslet, Beans, Shells, and Molluscs” pattern and a “Sweets” pattern which were identified in the second trimester only; and a “Pome, Berry and Melon fruits” pattern and a “Citrus” pattern which were identified only in the third trimester. The authors used the Bayley-III Screening Test to measure infant neurodevelopment at 12 months of age. Lv et al. (29) assessed neurodevelopment across five domains: cognition, expressive communication, receptive communication, fine motor, and gross motor. They found that a higher score on the “Aquatic products, Fresh vegetables and Homonemeae” pattern in the second trimester was significantly associated with a reduced risk of infants being deemed non-competent in cognitive development and gross motor development. Higher scores on the “Aquatic products and Homonemeae” pattern in the third trimester were also associated with a reduced risk of non-optimal cognitive development and receptive communication development. Higher scores on the “Nut” pattern in the second trimester was associated with a reduced risk of infants being non-competent in expressive communication. The dietary patterns consumed by pregnant women in our study differed substantially from the dietary patterns identified in Lv et al. (29). Notably, the pregnant women included in Lv et al. (29) study resided in China's eastern Jiangsu province, whereas our study participants were from Chongqing, in Southwestern China. Liu et al. (30) has demonstrated that maternal dietary patterns during pregnancy can vary significantly based on geographical variation, even within the same province. Despite different dietary patterns identified in each of the studies, both studies found a significant association between maternal dietary pattern consumption in pregnancy with aspects of motor development at 12 months of age.

The mechanisms driving these associations are not well understood. Associations between maternal dietary patterns and infant neurodevelopment have been hypothesized to be due to macronutrient under/overconsumption or micronutrient deficiencies in pregnancy (31); oxidative stress during pregnancy as a result of higher saturated fat consumption (32); and/or the ratio of omega-3:omega-6 fatty acid intake during pregnancy (32). On the contrary, Jacka et al. (33) highlighted that studies in animal models have demonstrated a relationship between maternal high sugar/fat diets and reduced infant brain plasticity, independent of nutrient deficiencies (33, 34) and maternal dams on a high fat diet had impaired child-rearing practices which may partly explain the relationship between maternal diet and infant brain development (35). More recently, evidence has emerged in humans to suggest that the mechanisms linking maternal dietary pattern consumption to infant neurocognitive and behavioral development may be via epigenetic modifications occurring *in utero*. House et al. (36) investigated the relationship between adherence to a Mediterranean-style dietary pattern in pregnancy and behavioral outcomes in infants, and explored the role of differentially methylated regions (DMRs) regulating genomically imprinted genes in explaining these associations. House et al. (36) found that adherence to the Mediterranean-style dietary pattern in pregnancy was associated with altered methylation patterns of the MEG3, SGCE/PEG10, PLAGL1 and IGF2 imprinted control regions (ICRs). The observed alterations to methylation patterns were also found to be associated with the infant behaviors related to adherence to the Mediterranean-style dietary pattern (36).

### Maternal Dietary Patterns and Pregnancy Outcomes

#### Gestational Diabetes Mellitus and Preeclampsia

Despite previous studies finding associations between maternal dietary patterns and the development of GDM and preeclampsia (10, 11, 37–39), no significant associations were found in our study. Results from systematic reviews have consistently found that women consuming a healthy dietary pattern in pregnancy (high in vegetables, fruits, legumes, fish and whole grains and low in meat, processed food and sugar-sweetened foods) had lower odds of developing pre-eclampsia and GDM (37–39).

There are some potential explanations for the disparity between our findings and others published. Firstly, it is possible that publication bias may have influenced the results published. Publication bias recognizes that the publication of positive findings are strongly favored, and therefore many non-significant findings may not have been disseminated or archived. Secondly, it is possible that the dietary patterns in early pregnancy are less of a predictor of GDM and preeclampsia than dietary patterns followed in the second and third trimesters, which was beyond the scope of this manuscript. Previous studies have investigated dietary patterns at varying times in pregnancy, however, many reporting significant findings predominantly investigated maternal dietary patterns in later pregnancy (7, 10, 11, 40, 41).

#### Gestational Weight Gain

Higher scores on the FPV-based dietary pattern were associated with higher gestational weight gain. Participants in the highest tertile of FPV dietary pattern score had the highest energy consumption (calories, Table 3). Energy consumption has been found to be related to gestational weight gain (42) and a separate study also partially accredited energy intake for the relationship observed between a “Western” dietary pattern and increased level of gestational weight gain (43). However, other studies investigating dietary patterns and their relation to gestational weight gain have failed to report or adjust for energy intake [e.g., (44)]. Therefore, it is difficult to ascertain whether significant associations reported previously are due to the dietary pattern itself, or if they are at least in part explained by energy intake.

#### Placental Weight

In addition to the relationship with psychomotor development, we found higher scores on the PSO-based dietary pattern to be associated with lower placental weight in our study. The placenta is crucial for the exchange of oxygen and nutrients to the fetus and for the removal of waste products. The placenta also has an endocrine role–producing hormones and eicosanoids during pregnancy such as estrogen, progesterone, and prostaglandins which are important for maintaining the pregnancy and preparing maternal tissues for delivery. Previous studies have found lower placental weight to be associated with reduced fetal growth and lower birthweight (45, 46), and Misra et al. (47) found a small but significant relationship between placental weight and IQ at 7 years in males. Our study finding suggests that placental development and therefore function may have been compromised in the women who were consuming a dietary pattern higher in pasta, sweetened beverages, oils and condiments, and fast food. Godfrey et al. (46) found that higher intake of carbohydrates in early pregnancy was associated with reduced placental weight and birthweight in their study (46). Carbohydrate intake (as a percentage of energy) was higher in participants in our study who scored in the top tertile of PSO-based dietary pattern consumption. However, the direct relationship between carbohydrate intake and placental weight was not significant in our study (analyses not shown).

In line with our hypothesis (i.e., that epigenetic modifications resulting from dietary pattern exposure may explain the relationship between dietary pattern consumption and infant neurodevelopment), other studies have shown both PEG10 and IGF2 to also be associated with placental growth and development (48, 49).

### Maternal Dietary Patterns and Infant Outcomes

#### Preterm Birth

No significant associations between dietary pattern consumption and preterm birth were found in this study. Previous studies have found significant associations between maternal dietary patterns and preterm birth (38, 50–53), and findings from a large systematic review and meta-analysis pooling studies prior to 2018 found that consumption of healthy dietary patterns (characterized by high intakes of vegetables, fruits, wholegrains, low-fat dairy, and lean protein foods) were associated with lower risk of preterm birth (OR for top compared with bottom tertile: 0.79; 95% CI: 0.68, 0.91; I2 = 32%) (9). Similar to the maternal dietary pattern studies investigating preeclampsia and GDM as outcomes, a large number of studies which identified significant associations were conducted later in pregnancy, and this might explain why we did not see a significant association in our study. Additionally, women with a previous pregnancy complication resulting in delivery before 32 weeks of gestation were not eligible for inclusion in the CLIMB study. Therefore, the CLIMB cohort may have had a sample of women who were at a lower risk of preterm delivery than the general population of pregnant women.

#### Small-For-Gestational-Age, Large-For-Gestational-Age, and Macrosomia

Maternal dietary pattern consumption in our study was not significantly associated with having a SGA or LGA baby or a baby with macrosomia. These findings are in line with the outcome from the large systematic review and meta-analysis which pooled the results of 14 studies (ten with SGA as an outcome, four with LGA as an outcome) and found that dietary pattern associations with SGA and LGA were inconsistent (9).

#### Body Composition

Maternal dietary pattern consumption was found to be associated with infant body composition at 6 weeks of age. Scores on the PSO-based dietary pattern were positively associated with infant tricep skinfold thickness at 6 weeks of age. Although the PSO-based dietary pattern was not necessarily a high-energy pattern (i.e., consumers in the top tertile had lower average kilocalorie consumption than those in the top tertile of the FPV-based dietary pattern), the PSO-based dietary pattern was more energy-dense (measured as ratio of kilocalories consumed to total grams of food consumed; data not shown). An energy-dense diet may also be reflective of a lower quality diet in terms of micronutrient composition, although we did not have reliable micronutrient intake data in this study to draw firm conclusions on the dietary quality.

A study conducted in a large Singaporean cohort found that a maternal dietary pattern high in fruit, vegetables, and white rice and low in fast food items and flavored rice was associated with lower infant tricep skinfold thickness from birth through 54 months of age (54). A study in the United States found a pattern high in poultry, nuts, cheese, fruits, whole grains, added sugars, and solid fats to be associated with a higher amount of newborn fat-free mass, but not fat mass or adiposity (55). They also found a pattern high in eggs, starchy vegetables, solid fats, fruits, and non-whole grains, and low in dairy foods, dark-green vegetables, and whole grains to be associated with a greater infant fat mass and adiposity (55). These examples demonstrate that although studies have found significant associations between maternal dietary patterns and infant body composition, the food components within the dietary patterns differ between cohorts, making comparisons between studies challenging.

### Strengths and Limitations

To our knowledge, this study is the first to report a relationship between maternal dietary patterns in early pregnancy and infant neurodevelopment in a Chinese cohort. We used a large cohort consisting of more than 1,000 mother-infant dyads from Chongqing, China. Associations between the maternal dietary patterns and a comprehensive range of pregnancy and infant outcomes were also investigated in this study.

Another strength of our study is that dietary patterns were derived using an *a posteriori* approach. An *a posteriori* data-driven approach to dietary pattern analysis has the advantage that findings derived from these analyses are representative of what is eaten within the population of interest. Therefore, findings have substantive value when considering their ability to be translated into relevant and meaningful population recommendations. Recommendations that require populations to make a slight shift in their current dietary patterns may increase compliance/adherence when compared to recommending a switch to an unfamiliar diet that may not be as culturally appropriate, accessible, or cost-effective.

Although our findings are statistically significant, they may not be particularly clinically significant. It is generally accepted that scores 2SD or more below the mean on the psychomotor development index (30 points below the mean of 100) are indicative of neurodevelopmental delay (56). Whereas, our findings showed a reduction of 1.276 points on the standardized PDI per SD increase in PSO score. This is not unexpected, as it is known that many factors can affect psychomotor development and therefore effects are likely to be cumulative, rather than due to one factor in isolation. In addition, our sensitivity analysis uncovered that the dietary pattern associations were influenced by women with the most extreme observations in dietary pattern scores, despite those observations being biologically plausible. Future studies may also benefit from adjusting for additional factors such as maternal IQ, which was not collected in this study.

A limitation of our study is that the FFQ was not sensitive enough to accurately measure micronutrient intake, and the Chinese food composition tables do not have complete micronutrient levels for all foods. Therefore, it was deemed inappropriate to assess micronutrient intake from the dietary data collected. As a result, we were unable to assess micronutrient intake across the tertiles of the dietary patterns consumed in our cohort. Future studies should consider assessing micronutrient status from biological measures where possible, if micronutrient composition data is not available.

Evidence also suggests that maternal education may be an important factor in determining dietary pattern selection in pregnancy as well as infant neurodevelopment. In a study of 7,462 women in Northwest China, it was found that pregnant women with high “balanced” pattern scores (a pattern high in meats, fungi, vegetables, fish, dairy, legumes, eggs, nuts, fast foods, and fruits) tended to be better educated (57). Our dietary pattern associations were adjusted for maternal education and family income to reduce the potential risk of confounding.

## Conclusions

In conclusion, we found a negative association between maternal consumption of the PSO-based dietary pattern and infant PDI scores at 12 months of age. This association was sensitive to extreme (but biologically plausible) scores on the PSO-based dietary pattern but the trend remained after sensitivity analysis (*P* = 0.085). This study is the first to report an association between maternal dietary patterns in early pregnancy and infant neurodevelopment in a Chinese cohort.

## Data Availability Statement

Data described in the manuscript can be made available upon reasonable request to the corresponding authors, pending application and approval.

## Ethics Statement

The studies involving human participants were reviewed and approved by Ethics Committee of Chongqing Medical University (#2014034). The patients/participants provided their written informed consent to participate in this study.

## Author Contributions

JdS, KB, CC, H-BQ, HZ, and PB designed the research. Y-YX, T-LH, and JdS conducted the research. JdS analyzed the data, with assistance from KB, MJ, and JC. JdS wrote the paper. All authors read and approved the final manuscript.

## Funding

This study was financially supported by a Joint Health Research Council New Zealand–National Science Foundation of China Biomedical Research Fund (19/804). JdS Postdoctoral Fellowship was funded by Lottery Health New Zealand. The CLIMB randomized controlled trial (which showed no significant difference between any of the intervention/control arms) was supported in part by the New Zealand Primary Growth Partnership Post Farmgate Dairy Programme, funded by Fonterra Co-operative Group Ltd., New Zealand and the New Zealand Ministry for Primary Industries. The funders were not involved in the design of this study, subsequent analysis, or interpretation of the findings presented in this manuscript.

## Conflict of Interest

JC serves as an occasional consultant to several maternal and infant food companies. The remaining authors declare that the research was conducted in the absence of any commercial or financial relationships that could be construed as a potential conflict of interest.

## Publisher's Note

All claims expressed in this article are solely those of the authors and do not necessarily represent those of their affiliated organizations, or those of the publisher, the editors and the reviewers. Any product that may be evaluated in this article, or claim that may be made by its manufacturer, is not guaranteed or endorsed by the publisher.
