# Supplementary material for: An Investigation of the Relationship Between Dietary Patterns in Early Pregnancy and Maternal/Infant Health Outcomes in a Chinese Cohort
Source: Front Nutr. 2022 Apr 22;9:775557. doi: 10.3389/fnut.2022.775557 (PMC9075413; doi:10.3389/fnut.2022.775557)
Supplement: Supplementary file 1 [file Data_Sheet_1.docx]

**Online Supporting Material**

Supplemental Table 1. Regression analyses of dietary pattern scores and pregnancy outcomes

|  | **β (95% CI)^1^; OR (95% CI)^2^** | **P-value** |
| --- | --- | --- |
| **Preterm birth^2^ (N=965)** |  |  |
| *FPV-based dietary pattern* |  |  |
| Unadjusted model | 1.060 (0.831, 1.353) | 0.637 |
| Adjusted model | 1.130 (0.818, 1.561) | 0.458 |
| *PSO-based dietary pattern* |  |  |
| Unadjusted model | 1.059 (0.885, 1.267) | 0.531 |
| Adjusted model | 1.165 (0.877, 1.546) | 0.291 |
| **Preeclampsia^2^ (N=962)** |  |  |
| *FPV-based dietary pattern* |  |  |
| Unadjusted model | 1.262 (0.847, 1.879) | 0.253 |
| Adjusted model | 1.106 (0.665, 1.838) | 0.698 |
| *PSO-based dietary pattern* |  |  |
| Unadjusted model | 0.943 (0.558, 1.593) | 0.826 |
| Adjusted model | 0.909 (0.499, 1.654) | 0.755 |
| **Gestational diabetes mellitus (GDM)^2^ (N=1261)** |  |  |
| *FPV-based dietary pattern* |  |  |
| Unadjusted model | 0.914 (0.810, 1.033) | 0.150 |
| Adjusted model | 1.033 (0.879, 1.214) | 0.689 |
| *PSO-based dietary pattern* |  |  |
| Unadjusted model | 1.009 (0.893, 1.139) | 0.891 |
| Adjusted model | 0.961 (0.815, 1.133) | 0.636 |

*Models adjusted for CLIMB treatment group, offspring sex, mother’s education level, age and BMI, family income, ethnicity, energy intake, and other dietary pattern*

*CI – confidence intervals; FPV- Fish, poultry, and vegetables; PSO- Pasta, sweetened beverages, oils and condiments*

**P<0.05*

*^1^Linear regression*

*^2^Logistic regression*

Supplemental Table 2. Regression analyses of dietary pattern scores and infant outcomes

|  | **β (95% CI)^1^; OR (95% CI)^2^** | **P-value** |
| --- | --- | --- |
| **Gestational age at delivery^1^ (N=1255)** |  |  |
| *FPV-based dietary pattern* |  |  |
| Unadjusted model | -0.212 (-0.865, 0.441) | 0.524 |
| Adjusted model | 0.061 (-0.722, 0.844) | 0.878 |
| *PSO-based dietary pattern* |  |  |
| Unadjusted model | -0.185 (-0.799, 0.430) | 0.555 |
| Adjusted model | -0.640 (-1.523, 0.243) | 0.155 |
| **Small for gestational age (SGA)^2^ (N=1231)** |  |  |
| *FPV-based dietary pattern* |  |  |
| Unadjusted model | 1.184 (0.895, 1.564) | 0.236 |
| Adjusted model | 1.119 (0.775, 1.614) | 0.549 |
| *PSO-based dietary pattern* |  |  |
| Unadjusted model | 0.870 (0.569, 1.328) | 0.517 |
| Adjusted model | 0.937 (0.607, 1.445) | 0.767 |
| **Large for gestational age (LGA)^2^ (N=1231)** |  |  |
| *FPV-based dietary pattern* |  |  |
| Unadjusted model | 1.265 (1.058, 1.513) | 0.010* |
| Adjusted model | 1.222 (0.963, 1.552) | 0.100 |
| *PSO-based dietary pattern* |  |  |
| Unadjusted model | 0.986 (0.814, 1.195) | 0.888 |
| Adjusted model | 0.860 (0.631, 1.172) | 0.340 |
| **Macrosomia^2^ (N=1231)** |  |  |
| *FPV-based dietary pattern* |  |  |
| Unadjusted model | 1.271 (0.994, 1.624) | 0.056 |
| Adjusted model | 1.265 (0.913, 1.753) | 0.158 |
| *PSO-based dietary pattern* |  |  |
| Unadjusted model | 1.065 (0.887, 1.279) | 0.498 |
| Adjusted model | 0.955 (0.653, 1.394) | 0.807 |
| **Ponderal index at birth^1^ (N=1217)** |  |  |
| *FPV-based dietary pattern* |  |  |
| Unadjusted model | -0.016 (-0.034, 0.002) | 0.084 |
| Adjusted model | -0.011 (-0.031, 0.009) | 0.283 |
| *PSO-based dietary pattern* |  |  |
| Unadjusted model | 0.001 (-0.022, 0.024) | 0.922 |
| Adjusted model | -0.004 (-0.027, 0.019) | 0.754 |
| **Subscapular skinfold thickness at six weeks^1^ (N=744)** |  |  |
| *FPV-based dietary pattern* |  |  |
| Unadjusted model | -0.026 (-0.182, 0.130) | 0.743 |
| Adjusted model | 0.071 (-0.102, 0.245) | 0.420 |
| *PSO-based dietary pattern* |  |  |
| Unadjusted model | 0.175 (-0.025, 0.375) | 0.085 |
| Adjusted model | 0.198 (-0.002, 0.398) | 0.052 |
| **Mid-arm circumference at six weeks^1^ (N=744)** |  |  |
| *FPV-based dietary pattern* |  |  |
| Unadjusted model | -0.003 (-0.100, 0.094) | 0.954 |
| Adjusted model | 0.058 (-0.050, 0.166) | 0.292 |
| *PSO-based dietary pattern* |  |  |
| Unadjusted model | 0.027 (-0.097, 0.152) | 0.665 |
| Adjusted model | 0.032 (-0.092, 0.157) | 0.609 |

*Models adjusted for CLIMB treatment group, offspring sex, mother’s education level, age and BMI, family income, ethnicity, energy intake, and other dietary pattern*

*CI – confidence intervals; FPV- Fish, poultry, and vegetables; PSO- Pasta, sweetened beverages, oils and condiments*

**P<0.05*

*^1^Linear regression*

*^2^Logistic regression*

Supplemental Table 3. Linear regression analyses of dietary pattern scores and pregnancy outcomes for sensitivity analysis

|  | **β (95% CI)** | **P-value** |
| --- | --- | --- |
| **Placental weight (N=996)** |  |  |
| *FPV-based dietary pattern* |  |  |
| Unadjusted model | -2.779 (-7.329, 1.771) | 0.231 |
| Adjusted model | -1.930 (-6.964, 3.103) | 0.452 |
| *PSO-based dietary pattern* |  |  |
| Unadjusted model | -6.149 (-12.687, 0.388) | 0.065 |
| Adjusted model | -5.841 (-12.363, 0.681) | 0.079 |
| **Gestational weight gain (N=1172)** |  |  |
| *FPV-based dietary pattern* |  |  |
| Unadjusted model | 42.473 (21.974, 62.972) | <0.001* |
| Adjusted model | 35.629 (11.666, 59.592) | 0.004* |
| *PSO-based dietary pattern* |  |  |
| Unadjusted model | 7.648 (-23.779, 39.075) | 0.632 |
| Adjusted model | -1.682 (-33.275, 29.910) | 0.917 |

*Models adjusted for CLIMB treatment group, offspring sex, mother’s education level, age and BMI, family income, ethnicity, energy intake, and other dietary pattern*

*CI – confidence intervals; FPV- Fish, poultry, and vegetables; PSO- Pasta, sweetened beverages, oils and condiments*

**P<0.05*

Supplemental Table 4. Regression analyses of dietary pattern scores and infant outcomes for sensitivity analysis

|  | **β (95% CI)^1^;OR (95%CI)^2^** | **P-value** |
| --- | --- | --- |
| **Large for gestational age (LGA)^2^ (N=1207)** |  |  |
| *FPV-based dietary pattern* |  |  |
| Unadjusted model | 1.277 (1.052, 1.549) | 0.013* |
| Adjusted model | 1.318 (1.058, 1.641) | 0.014* |
| *PSO-based dietary pattern* |  |  |
| Unadjusted model | 0.857 (0.623, 1.178) | 0.341 |
| Adjusted model | 0.814 (0.584, 1.135) | 0.225 |
| **Standardized scores on the Bayley Scales of Infant Development Psychomotor development index at 12 months (N=955)** |  |  |
| *FPV-based dietary pattern* |  |  |
| Unadjusted model | -0.255 (-1.260, 0.749) | 0.618 |
| Adjusted model | -0.253 (-1.368, 0.861) | 0.656 |
| *PSO-based dietary pattern* |  |  |
| Unadjusted model | -1.192 (-2.532, 0.147) | 0.081 |
| Adjusted model | -1.191 (-2.545, 0.164) | 0.085 |
| **Tricep skinfold thickness at six weeks (N=704)** |  |  |
| *FPV-based dietary pattern* |  |  |
| Unadjusted model | 0.175 (-0.122, 0.472) | 0.247 |
| Adjusted model | 0.136 (-0.210, 0.483) | 0.439 |
| *PSO-based dietary pattern* |  |  |
| Unadjusted model | 0.187 (-0.256, 0.631) | 0.407 |
| Adjusted model | 0.093 (-0.364, 0.550) | 0.689 |

*Models adjusted for CLIMB treatment group, offspring sex, mother’s education level, age and BMI, family income, ethnicity, energy intake, and other dietary pattern*

*CI – confidence intervals; FPV- Fish, poultry, and vegetables; PSO- Pasta, sweetened beverages, oils and condiments*

**P<0.05*

*^1^Linear regression*

*^2^Logistic regression*
